# Impact of Grape Harvest Time on Wild Yeast Biodiversity and Its Influence on Wine Fermentation

**DOI:** 10.3390/microorganisms13122836

**Published:** 2025-12-13

**Authors:** Mercè Sunyer-Figueres, Daniel Fernández-Vázquez, Cristina Cuesta-Martí, Inés Horcajo-Abal, Carlos Sánchez-Mateos, Alba Domènech, Enric Nart, Victoria A. Castillo-Olaya, Immaculada Andorrà, Miquel Puxeu

**Affiliations:** 1 Centre Tecnològic del Vi, Carretera de Porrera Km. 1, 43730 Falset, Spain; merce.sunyer@vitec.wine (M.S.-F.); daniel.fernandez@vitec.wine (D.F.-V.); ines.horcajo@vitec.wine (I.H.-A.); carlos.sanchez@vitec.wine (C.S.-M.); alba.domenech@vitec.wine (A.D.); enric.nart@vitec.wine (E.N.); victoria.castillo@vitec.wine (V.A.C.-O.); miquel.puxeu@vitec.wine (M.P.); 2 Department of Anatomy and Neuroscience, University College Cork, T12 K8AF Cork, Ireland; ccuestamarti@outlook.com; 3 APC Microbiome Ireland, University College Cork, T12 YN60 Cork, Ireland

**Keywords:** ripening, *Torulaspora delbrueckii*, *Hanseniaspora*, *Starmerella bacillaris*, climate change, autochthonous, spontaneous fermentations, ecology

## Abstract

Grape harvest time influences the berry composition, which impacts the organoleptic wine characteristics. Winemakers monitor technological, phenolic, and aromatic grape maturity to decide the harvest date. Little is known about the evolution of yeast ecology of grapes during the maturation period. The microbiota involved in the fermentation impacts the wine composition and characteristics; therefore, changes in grape biodiversity could have an impact in fermentation kinetics and aroma compound formation. In this study, the yeast biodiversity of Grenache Noir and Carignan grapes from Montsant DO (Denomination of Origin) were analyzed at different ripening stages to assess how harvest time influences microbiota. The fermentation performance of the yeasts obtained was studied at both laboratory and pilot scales to evaluate the impact of these yeasts, both in pure and mixed cultures, on the wine’s chemical and aromatic composition as well as its sensory impact. The results indicated that early harvest favored a higher diversity of non-*Saccharomyces* species, whereas in more mature grapes, *Saccharomyces cerevisiae* species was dominant. The isolated strains of *Saccharomyces* differed in their fermentation performances, as well as ethanol content and acidity of wine. In general, they produced higher concentration of fermentative volatile compounds than a commercial strain. The non-*Saccharomyces* yeasts in mixed fermentations with the *S. cerevisiae* strains also impacted wine composition and characteristics, leading lower ethanol content or enhancing aromatic balance and sensory equilibrium. The results highlight that grape harvest timing influences microbial diversity and fermentative performance and thus should be considered to better determine the optimum harvest date and ensure consistent wine characteristics.

## 1. Introduction

The grape microbiome is a key factor that impacts the composition and aroma of the wine. The autochthonous biodiversity of the grape is linked to the microbial fingerprint responsible for the distinctive oenological properties of each region, referred to as the microbial “terroir” [1,2,3,4,5]. The microbiota of grapes depends on several factors such as climate, grape variety, vintage, vineyard characteristics, soil, and viticultural practices [1,3]. In this context, spontaneous fermentations are gaining attention for their potential effects on the organoleptic complexity of wine. However, spontaneous fermentations have been shown to present higher risk of slow or even stuck or sluggish fermentation [6], which can cause spoilage and therefore affect the stability and aromatic properties of wines. This can result in wines lacking desirable characteristics [7] and potential economic losses for wineries. For this reason, recent research has focused on studying the use of selected autochthonous microorganisms from each region as starter cultures to simulate spontaneous fermentation in a controlled way [8,9]. In fact, the search for autochthonous yeasts is experiencing increased attention not only to increase the aromatic complexity of wines, but also for the properties of some yeast strains to face today’s challenges of the oenological industry. Those challenges could be the higher ethanol content and lower acidity of wines caused by global warming [10,11].

The main yeasts initiating the spontaneous alcoholic fermentation are a diverse range of indigenous non-*Saccharomyces* yeasts, since they, together with other fungi, are the predominant microbiota in grapes [12]. Most non-*Saccharomyces* yeasts contribute to the early phases of the fermentation process until the ethanol concentration reaches 3–4% (*v*/*v*). After that, *Saccharomyces* yeasts dominate the middle and the end of alcoholic fermentation, due to several aspects: their higher fermentative capacity [13,14,15], growth rate, and tolerance to ethanol compared to well-adapted non-*Saccharomyces* species [13]. Traditionally, *Saccharomyces cerevisiae* was universally preferred to initiate the fermentation process [14,16]. However, recently oenologists have shown interest in using mixed cultures of *S. cerevisiae* and non-*Saccharomyces* to produce wines with different characteristics based on improved wine body, taste, and aroma. Non-*Saccharomyces* yeasts contribute to these qualities via the production of secondary metabolites or extracellular enzymes. *Saccharomyces*, on the other hand, provides high fermentative capacity and kinetics [9,17].

The grape harvest date is probably one of the most important decisions that winemakers take [18]. They must assess the appropriate moment of grape maturity that depends on different factors such as the quantity of sugars and acidity levels according to pH and total acidity (technology maturity), total and extractive polyphenols from skin and seed (phenolic maturity), and primary aromas and aromatic precursors (aromatic maturity), among others [19,20]. Certain studies have focused on analyzing the impact of harvest time on the final wine. Ramos et al. [21] reported an increased content of 3,4-dihydroxybenzoic when the harvest time was extended. Tian et al., 2009 observed that the harvest time is crucial for the evolution of phenolic acids and flavan-3-ols during fermentation, which is key to the phenolic content in the final wine [22].

However, little is known about how the communities of wine yeasts evolve in relation to harvest timing. The microbial composition on the surface of berries varies between non-damaged and damaged berries, where the concentration of nutrients increases. Grapemust is a highly nutritious environment for yeasts. However, there are some factors that restrict the presence of certain microorganisms, including pH, high osmotic pressure, low water activity, and the presence of sulfur dioxide [14].

Harvest timing is related to the composition of the grapes and could shape the biodiversity of the grape surface. Therefore, it could affect fermentation dynamics and the characteristics of the final wine. Identifying the optimal harvest time from a microbiological perspective could help to improve both the desired wine profile and fermentation performance. The present study aimed to elucidate how the grape surface microbiome evolves during the ripening process and to assess the fermentative potential of the yeast microbiota.

## 2. Materials and Methods

### 2.1. Spontaneous Fermentation

**Figure 1:**
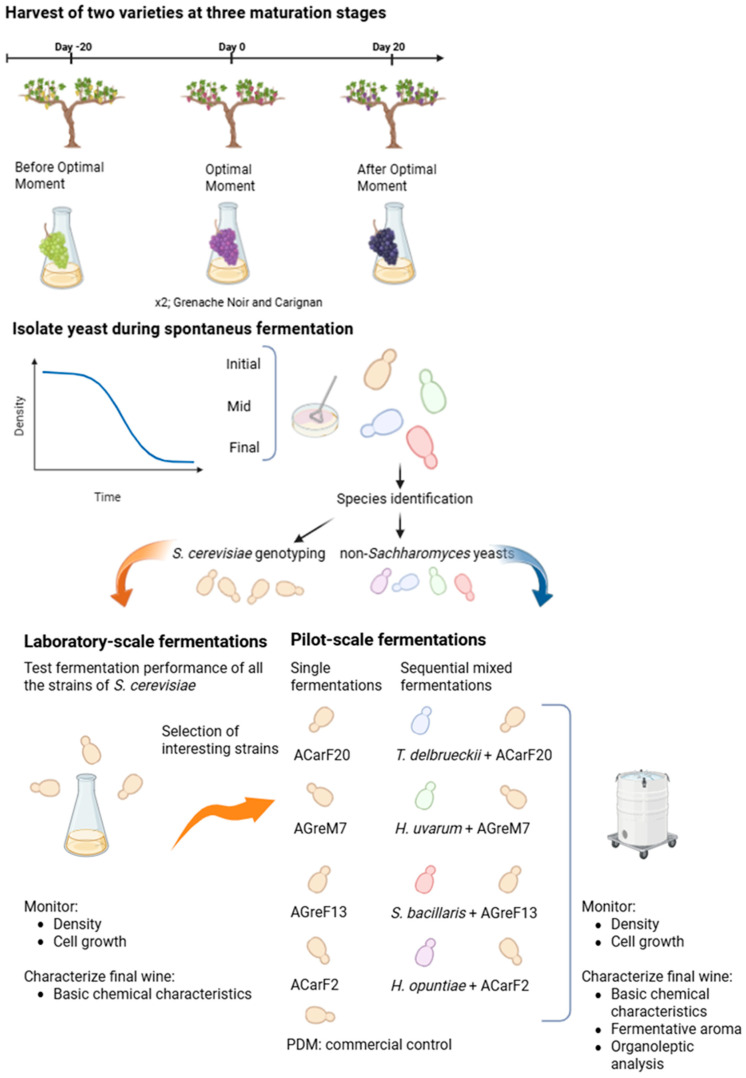
Experimental scheme illustrating the experimental methods described in Section 2.

A Montsant DO (Denomination of Origin) vineyard was selected for this study for the collection of Grenache Noir and Carignan grapes. The vineyard was located in Falset (Catalonia, Spain) at an altitude of 365 m above sea level, with a southeast orientation, an annual rainfall of 300 L, and decomposed granite soils. Fifty grape berries per variety and ripening stage were aseptically collected by hand in each vineyard from 10 randomly selected grapevines to ensure coverage of the entire vineyard area. The maturation points were chosen according to the optimum harvest stage defined by the viticulturist for harvest, which was based mainly on technological maturity (Table 1). The authors acknowledge that “optimum” is a subjective term that differs for each viticulturer and depends on the wine typology. Nevertheless, this term is used throughout the article for enhancing understanding. Three harvest times relative to the optimum one were selected: 20 days before, at the optimum harvest date determined by the winemaker, and 20 days after (Figure 1).

The grapes were transported in sterile bags to the laboratory in refrigerated conditions. In the laboratory, the grapes were aseptically pressed to obtain the must, which was placed in 500 mL sterile flasks, and the density was measured. Spontaneous fermentations were monitored through the analysis of temperature and must density with an electronic densitometer Densito^TM^ 30px (Mettler Toledo, Columbus, OH, USA), along with total and viable yeast counts, as explained in Section 2.3. Finally, the yeasts were isolated at 3 different fermentation stages: the beginning of alcoholic fermentation (I; density > 1.080 g/cm^3^), the middle point of alcoholic fermentation (M; density 1.040–1.060 g/cm^3^), and the end of the spontaneous fermentation (F; density < 1.010 g/cm^3^) (Figure 1).

### 2.2. Yeasts Isolation

For each ripening stage (before the optimum moment (B), at the optimum (O), and after the optimum (A)) and for each fermentation stage (initial (I), mid (M), and final (F)), must aliquots were seeded in solid YPDA media plates (Yeast Peptone Dextrose Agar; composition: 2% glucose, 2% peptone, 1% yeast extract, 2% agar, *w*/*v*; Panreac, Barcelona, Spain) and incubated at 27 °C for 48 h. In order to identify the yeasts present at each fermentation and harvesting time point, 24 colonies from each plate were randomly picked and inoculated into tubes containing liquid YPD medium (glucose 2%, peptone 2%, yeast extract 1% *w*/*v*; Panreac, Barcelona, Spain), which were incubated at 27 °C for 24 h with agitation. Once grown, cultures were centrifuged to obtain the pellet for subsequent DNA extraction, which was performed following the protocol described by Querol et al. [23]. All strains obtained in this study were stored in the laboratory and cryopreserved in glycerol at <−70 °C.

### 2.3. Yeasts Identification and Genotyping

After DNA extraction, the identification of yeasts at the species level was carried out via PCR-RFLP analysis of 5.8 S-ITS rDNA using primers ITS1 (5′-TCCGTAGGTGAACCTGCGG-3′) and ITS4 (5′-TCCTCCGCTTATTGATATGC-3′) (Biomers, Ulm, Germany), according to Esteve-Zarzoso et al. [24]. Afterwards, PCR products were separated by 1.6% agarose gel electrophoresis. Additionally, a sample of the respective PCR product was digested with the restriction enzymes *Dde*I and *Hinf*I and then separated by 3% agarose gel electrophoresis (using RedSafe Nucleic Acid Staining Solution (20.000X) (iNtRON. Biotechnology, Seongnam, Republic of Korea) and HyperLadder 100 bp (Bioline, London, UK)). The results of the electrophoresis of both PCR products before and after digestion with restriction enzymes were compared with previous studies using a similar procedure.

A sample of the PCR product (before digestion with restriction enzymes) was sent for sequencing to Macrogen Spain (Madrid, Spain) in order to confirm the identity of the yeasts. The sequences obtained were aligned using the GenBank BLAST tool (https://blast.ncbi.nlm.nih.gov/Blast.cgi (accessed on 29 July 2020)).

After yeast identification, *S. cerevisiae* isolates were genotyped (i.e., genetically characterized) by the analysis of the inter-delta regions with the primers δ12 (5′-TCAACAATGGAATCCCAAC-3′) and δ21 (5′-CATCTTAACACCGTATATGA-3′) (Biomers, Ulm, Germany), as described by Legras and Karst [25]. Prior to PCR amplification, the quantification and quality analysis of the DNA of the *S. cerevisiae* isolate was analyzed using the BioDrop μLite technique from Isogen Life Science (Servicios Hospitalarios S.L, Barcelona, Spain). Afterwards, PCR products were separated by 1.6% agarose gel electrophoresis using the RedSafe Nucleic Acid Staining Solution (20.000X) (iNtRON. Biotechnology, Seongnam, Republic of Korea) and HyperLadder 1Kb (Bioline, London, UK).

### 2.4. Laboratory Scale Fermentations of S. cerevisiae Strains

The fermentative performance of some of the strains of *Saccharomyces cerevisiae* was tested by monitoring the fermentation of red must in duplicate at laboratory scale (Figure 1). The must was obtained by diluting concentrated sterile red must (with an initial Brix of 65–69°) (Concentrats Pallejà S.L., Riudoms, Tarragona, Spain) to obtain a must with a Brix grade of 23.6 for the Carignan strains and 27.0 Brix grade for the Grenache Noir strains. The chemical characterization of the must was performed, as described in Section 2.6, and the results are presented in Table A1. The cryopreserved yeasts were cultivated on YPDA plates at 28 °C for 2–4 days. When needed, the yeasts were pre-incubated in 10 mL of YPD medium at 25 °C in a rotatory shaker at 100 rpm for 48 h and then inoculated into 20 mL of YPD for 24–48 h to obtain an inoculum concentration of 10^7^–10^8^ cells/mL. Must flasks were inoculated from these pre-cultures to obtain a yeast concentration of 2.0 × 10^6^ cells/mL. The flasks were then incubated at a controlled and constant temperature (25 °C) without agitation. Fermentations were monitored as described in Section 2.5, and the basic oenological parameters of the final wines were analyzed as explained in Section 2.6.

### 2.5. Fermentation Monitoring

The fermentation process was monitored by measuring the density using an electronic densimeter (Excellence D5, Mettler Toledo, Barcelona, Spain) until the sugar concentration was lower than 2 g/L, measured as described in Section 2.6. Furthermore, the yeast population was monitored by counting the total number of cells by microscopy using a Thoma chamber from BRAND GmbH + CO KG (Wertheim, Germany), total viable yeasts using YPDA medium, and non-*Saccharomyces* viable yeasts by plating in Lysine agar (Oxoid, Hampshire, UK), a selective medium that inhibits the growth of *Saccharomyces* spp. The population of *S. cerevisiae* at each time point was calculated by subtracting the colonies that grew on Lysine agar from the colonies that grew on YPDA [26].

### 2.6. Oenological Characterization of Musts and Wines

The oenological parameters of all musts and wines were measured according to the methods recommended by the *Compendium of International Methods of Analysis—Organization of Vine and Wine* [27]. An enzymatic technique (Y200 Biosystems, BioSystems S.A., Barcelona, Spain) was used to analyze yeast assimilable nitrogen (YAN), glucose–fructose, L-malic acid, and L-lactic acid. A wine ScanSO_2_ (Foss, Barcelona, Spain) was used to analyze alcohol content, pH, total acidity (TA) concentration expressed as tartaric acid, volatile acidity concentration expressed as acetic acid, and Brix grade. The ratio of yield of sugar transformation into ethanol in each condition was calculated by dividing the ethanol concentration (g/L) in the final wine by the concentration of glucose–fructose consumed. The concentration of ethanol was calculated by multiplying the acquired alcoholic degree by 7.894, as explained in Monnin et al. [28].

### 2.7. Microvinifications at Pilot Scale

The *S. cerevisiae* strains with interesting fermentation characteristics were chosen for testing its performance at higher volumes (on a pilot scale) to achieve conditions more similar to those in wineries. Moreover, the potential of some of the non-*Saccharomyces* strains was also tested by sequential inoculation with *S. cerevisiae*. For this, single and mixed fermentations were performed in duplicate at pilot scale (Figure 1).

Must was obtained from Trepat grapes harvested in the 2023 vintage, with characteristics similar to those listed in Table 1 for non-mature must. Grapes were hand-picked and placed into 15 kg boxes, before being transported to the experimental winery of VITEC (Wine Technology Center, Tarragona, Spain) at 4 °C. Grapes were then destemmed, crushed, and transferred into 50 L steel tanks. The must was analyzed for its basic chemical and microbiological parameters. The results of the chemical characterization are summarized in Table A1.

Microvinifications were performed at the experimental winery using 50 kg of Trepat grapes, which were fermented at a controlled and constant temperature (22 °C). Single fermentations were performed using the strains isolated during this study (ACarF20, ACarF2, AGreF13, and AGreM7). Mixed fermentations were conducted by inoculating non-*Saccharomyces* strains isolated during this study (OGreM1, ACarM21, ACarI7, and BCarM1, corresponding to *Torulaspora delbrueckii*, *Hanseniaspora opuntiae*, *Starmerella bacillaris* (synonym *Candida zemplinina*), and *Hanseniaspora uvarum*, respectively). 48 h later *S. cerevisiae* was inoculated, different strains of *S. cerevisiae* were used for each condition. A control condition was performed using a single fermentation with the commercial strain of *S. cerevisiae* Fermivin PDM (Oenobrands, Montpellier, France). All the *S. cerevisiae* and no-*Saccharomyces* strains were inoculated at 2.0 × 10^6^ cells/mL from a culture produced, as explained in Section 2.4 (Figure 1).

The must was supplemented at different time points during alcoholic fermentation with: Actimax Varietal™ (Agrovin, Alcázar de San Juan, Spain) at 30 g/hL at the beginning, Actimax Plus™ (Agrovin, Spain) at 20 g/hL when density reached 1.040 g/cm^3^, and SB Evolution™ (Agrovin, Spain) at 10 g/hL when density reached 1.020 g/cm^3^. The fermentations were monitored as described in Section 2.5, and the basic oenological parameters of the final wines were analyzed as explained in Section 2.6. Wines were bottled and stored until they were tested in the same month. For all wines obtained, wine fermentative compounds were determined as described in Section 2.8. The organoleptic characteristics were evaluated as described in Section 2.9.

### 2.8. Determination of Wine Aroma Compounds

The volatile aroma compounds of all pilot-scale microvinifications were analyzed by gas-chromatography GC 7890A (Agilent Technologies, Santa Clara, CA, USA) coupled to a Quadrupole Mass Detector 5975C MSD [29]. Briefly, samples (10 mL) were placed in 20 mL headspace vials together with 2.7 g NaCl and 100 μL of 2-octanol (1.000 ppm) as internal standard. The column used was a DB-WAX UI (60 m × 0.25 mm × 0.25 μm, Agilent Technologies). A constant flow of 1.6 mL/min of helium was used as carrier gas to a pressure of 25 psi. The results for the volatile compounds were semi-quantitative data in relation to the response provided by the internal standard (2-octanol). All analyses were performed in triplicate. In order to evaluate the contribution of the aromatic compounds to the aroma of the wine, the odorant activity value (OAV) was calculated for each compound. This parameter is calculated as the ratio between the concentration of each compound and its corresponding perception threshold [30,31]. If the calculated OAV for a certain compound is greater than the unity, that compound can be considered as an active aroma [29,32].

### 2.9. Organoleptic Evaluation

The quantitative descriptive analysis was performed by a trained tasting panel following the ISO 8586:2023 [33] normative. The panel operates under sensory evaluating conditions aligned with the ISO 17025 standard [34], which ensure reliability and robustness of the results, as the judges were trained and evaluated to provide precise, reproducible, and reliable evaluations. Thus, a total of 10 wines were blindly tasted by a gender-balanced panel of 5 judges in the normalized ISO 8589:2007 [35] room of VITEC. Wine sensory evaluation was classified into different attributes, including color intensity, aroma (intensity and profile), and flavor (acidity, tannic intensity, astringency, dryness, unctuosity, persistence, and burning). Among the aroma profiles, fruity aromas (red fruit, black fruit, and candied fruit aromas), floral, balsamic, and lactic aromas were considered interesting attributes. Panelists were required to rate the intensity of the wine parameters using a five-point scale (0 = absence, 5 = maximum intensity). Data were collected with tablets using Compusense^®^ Cloud software (Version 25.0.30595, Compusense Inc., Guelph, ON, Canada). Informed consent for participation was obtained from all subjects involved in the study [36].

### 2.10. Statistical Analysis in XLSTAT

The data were subjected to a one-way analysis of variance (ANOVA) and Tukey’s post hoc test to evaluate the effect of each fermentation. The results were considered statistically significant at a *p*-value < 0.05 using XLSTAT (Addinsoft, New York, NY, USA). Principal Component Analysis (PCA) was performed to visualize a 2D plot of the first two principal components (PCs) using XLSTAT Version 2016.01.26717 (Addinsoft, NY, USA). Normality and homogeneity of variance were assumed for the data used in these analyses [37].

## 3. Results and Discussion

### 3.1. Spontaneous Fermentations

**Figure 2:**
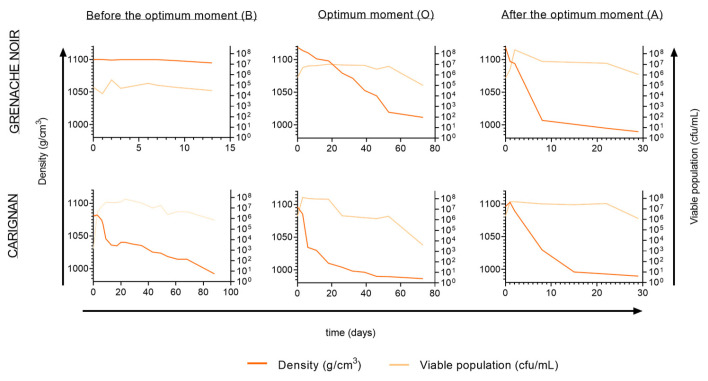
Evolution of spontaneous fermentations for Grenache Noir and Carignan grape varieties, harvested at the three maturity stages (B, before the optimum moment; O, at the optimum moment; A, after the optimum moment).

Different yields in terms of the yeast population of grapes were found at the various ripening stages analyzed. Before the optimum ripening moment, the lowest populations were observed for both Grenache (8 × 10^4^ cfu/mL) and Carignan (10^3^ cfu/mL). At the optimum moment and after the optimum moment, grape populations varied from 5 × 10^5^ cfu/mL for Grenache to 5 × 10^6^ cfu/mL for Carignan (Figure 2).

**Figure 3:**
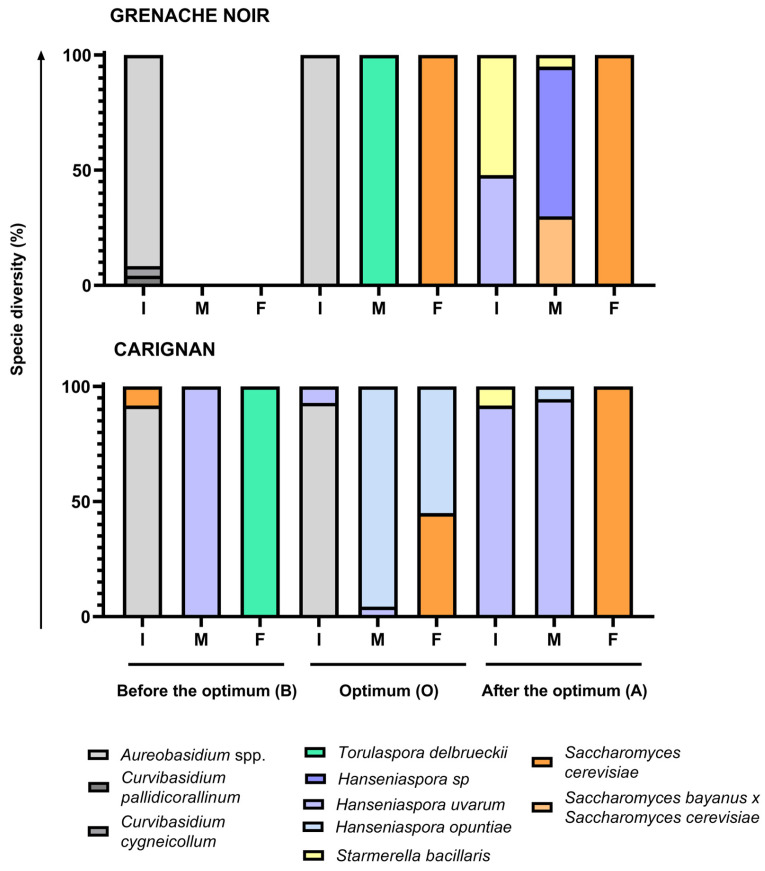
Diversity of species isolated and identified in the Carignan and Grenache Noir grape varieties according to the harvest time (B for before the optimum moment, O for the optimum moment, and A for after the optimum moment) and the stage of alcoholic fermentation (I for the start of fermentation, M for mid-fermentation, and F for the end of fermentation).

Spontaneous fermentations were monitored and considered as finished when sugar levels were <2 g/L. Although all fermentations followed different kinetics, they all reached yeast populations of around 5 × 10^7^ cfu/mL within the first few days of fermentation, as reported elsewhere [38,39]. However, fermentations using Grenache grapes harvested before the optimum moment remained at 10^5^ cfu/mL after 13 days, with a density above 1.095 g/cm^3^, indicating incomplete fermentation. In the case of the Carignan fermentation, the must from grapes harvested before the optimum moment showed a low fermentation rate, taking 88 days to consume all sugars. A grape microbial community dominated by fungi and bacteria, with an initial low yeast population, hinders the rapid initiation of fermentation and can result in a slower sugar consumption rate [38]. This situation likely occurred at the early ripening moment, where isolates from the first fermentation points were mainly identified as *Aureobasidium* and *Curvivasidium* genera (Figure 3).

**Table 1:** Chemical parameters of the musts at different grape states: B (before the optimum moment), O (optimum moment), and A (after the optimum moment).

Must Variety	Parameter	B	O	A
Grenache Noir	° Brix	23.6	27.2	27.6
	Potential alcohol (% *v*/*v*)	13.7	16.1	16.65
	Density (g/cm^3^)	1.100	1.118	1.119
Carignan	°Brix	19.6	22.7	24
	Potential alcohol (% *v*/*v*)	11	13	14
	Density (g/cm^3^)	1.082	1.096	1.100

For the optimum ripening moment, fermentation kinetics presented quicker sugar consumption. In the case of Grenache, yeast population did not reach 10^7^ cfu/mL, and the fermentation ultimately became stuck with 89.4 g/L of residual sugars. In the case of Carignan, the fermentation lasted 72 days but consumed all sugars. Musts obtained from grapes harvested after the optimum ripening moment for both varieties showed the best fermentation rate. All sugars were consumed by day 30, presenting a short lag phase at the beginning and a consistent fermentation rate. In this study, only the overmature ripening stage resulted in successful fermentations for both grape varieties. Fermentations with long durations are problematic for the wine industry, as they are often associated with sluggish or stuck fermentations that are difficult to restart. Multiple factors such as excessive use of sulfur dioxide, temperature changes, or dissolved oxygen availability can directly affect fermentation performance [6].

Normally, nitrogen depletion is one of the main reasons for slow fermentations [6,40] because yeasts need it to metabolize sugar. The glucophilic or fructophilic preference of different yeast species is also important, as it affects their capacity to fully remove sugars (considered as the sum of glucose and fructose) during long-lasting fermentations. *S. cerevisiae* prefers glucose [41,42,43], while other species such as *Zygosaccharomyces bailii* [44] or *T. delbrueckii* [45] are mainly fructophilic. When fermentation stops, the normal cellar practice is to reinoculate, but the addition of yeast must ensure that there is sufficient nitrogen and that the type of sugar will be consumed.

### 3.2. Yeasts Isolated During Spontaneous Fermentation

A total of 446 colonies were isolated and identified using the ITS-5.8 S rDNA PCR-RFLP technique. Of these, 308 were identified as yeasts, while the remainder were identified as fungi or bacteria. The most abundant fungi (apart from yeasts) found in both must varieties were *Aureobasidium* spp. It had an imposition of at least 90% at the initial fermentation point in musts harvested before the optimum moment and at the optimum harvest moment (Figure 3). Other fungi such as *Curvibasidium pallidicorallinum* and *Curvibasidium cygenicollum* were also identified at the initial stage of fermentation of Grenache Noir must harvested before the optimum ripening time. Only yeasts were identified in spontaneous fermentations of musts harvested after optimum harvest moment for both varieties. Populations of fungi other than yeast declined in all musts at the mid-fermentation point (density around 1.040–1.060 g/cm^3^). Yeast species were dominated in a different way in each case. These fungi are sensitive to the conditions of winemaking, particularly ethanol [46], which makes it difficult to find them at the mid-point of fermentation.

The fermentation of Grenache Noir must, harvested before the optimum moment, was stuck before reaching 1.040 g/cm^3^, so in the unique point sampled—the beginning of fermentation—no yeasts were found. For the Carignan must harvested before the optimum moment, non-*Saccharomyces* yeast was identified: *Hanseniaspora uvarum* at mid-fermentation (100%) and *Torulaspora delbrueckii* (100%) at the end of fermentation. Almost 10% of isolates were identified as *S. cerevisiae* at the initial point, but they were not detected again during the fermentation [47].

Regarding the optimum harvesting moment, yeasts imposed at mid-fermentation point, being *T. delbrueckii* (100%) in the case of Grenache Noir and *H. opuntiae* (95%) and *H. uvarum* (5%) in the case of Carignan. *S. cerevisiae* imposed at the end of fermentation in Grenache Noir (100%) but represented <50% of the population in Carignan, where *H. opuntiae* populations survived until the end. *T. delbrueckii* and some of the *Hanseniaspora* species identified were described as more resistant to ethanol than most of the non-*Saccharomyces* yeasts, tolerating ethanol contents up to 9% *v*/*v* in the case of *T. delbrueckii* [48]. This yeast was described as being able to maintain a respiratory metabolism under low oxygen conditions [49]. Despite having a slower growth and fermentation rate than *S. cerevisiae*, it can grow and impose sometimes in musts with a low population of stronger fermenters [50].

In the fermentations of musts harvested after the optimum moment, both grape varieties exhibited comparable yeast diversity, featuring *S. bacillaris* in combination with *H. uvarum* at the initial time point. At the mid-fermentation point, *H. uvarum* dominated, while *S. bayanus x S. cerevisiae* isolates were also identified in the case of Grenache Noir. By the end of fermentation, *S. cerevisiae* had become dominant. *Hanseniaspora uvarum* has been described by Mestre et al., 2019 as quite resistant to ethanol but also to SO_2_ and high sugar concentrations [51]. *S. bacillaris* exhibits resistance to very high sugar concentrations [52,53]. The high osmotolerance of these species may be related to the fact that they are found in the must with the higher Brix grade (Table 1). Moreover, *S. bacillaris* produces low alcohol content due to the production of other secondary metabolites such as pyruvic acid or glycerol [52,54]. It has also been described that this species is fructophilic, so it can consume fructose and impose more than other glucophilic yeasts at certain fermentation stages [45]. These species have also been described in the literature in spontaneous fermentations during the first stages of vinification [38,55].

Yeast biodiversity found was low for spontaneous fermentations, as a maximum of three species were identified in one single fermentation stage. In almost all fermentation stages from all harvest moments, there was only one dominant yeast species. Must fermentation itself is a selective process where low-represented yeast species can be selected under its specific fermenting conditions. This low biodiversity could be attributed to various viticultural or environmental factors that were not evaluated in this study. Vineyard management practices or climatic conditions prior to harvest could have significantly influenced the microbial composition of the grapes.

**Figure 4:**
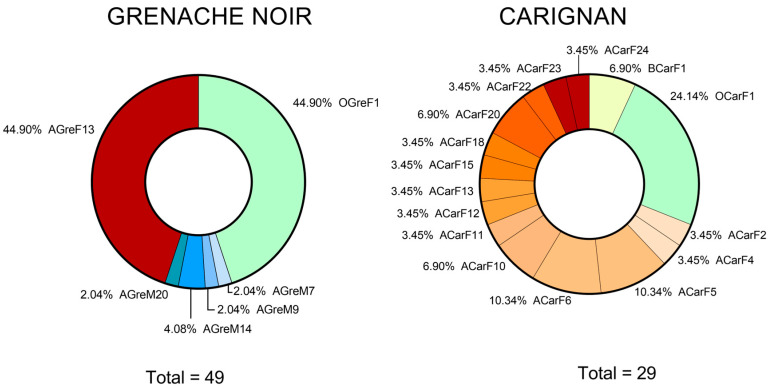
Abundance of *S. cerevisiae* strains from Carignan and Grenache Noir at different harvest moments and fermentation stages. The total represents the number of *S. cerevisiae* colonies isolated and genotyped for each variety. Letters in strain names identify different harvesting states (B for before the optimum moment, O for optimum moment, and A for after the optimum moment), grape varieties (Gre for Grenache Noir and Car for Carignan), and fermentation sampling stages (M for mid-fermentation and F for the final fermentation stage). Totals may not sum to 100% due to rounding.

The *S. cerevisiae* profiles identified in the Grenache Noir variety (6) were obtained at the optimum harvest time (one profile at the end point of fermentation) and after the optimum moment (four profiles at the mid-point and one profile at the end of fermentation) (Figure 4). The Carignan variety profiles (16) were obtained at the final stages of fermentation of musts harvested before the optimum moment and at the optimum moment (one profile each). Most profiles (14) were obtained after the optimum moment. Notably, all the *S. cerevisiae* isolates identified in the final stage of fermentations at the optimum moment belonged to the same profile. In the Grenache Noir must that was harvested after the optimum moment, four different profiles were found in the mid-point of fermentation. By the end of the fermentation, one strain dominated. For the Carignan must at the end of fermentation, several strains could be distinguished. The strain that adapts best to the must usually dominates during the fermentation due to its higher fermentation rate. In the present study, only one profile was detected for Grenache Noir at the end of fermentation, whereas several profiles could be identified for Carignan. These results are supported by other studies, in which the authors have described different behaviors for different *S. cerevisiae* consortia. In the study of Bedoya et al. [55], several strains of *S. cerevisiae* were inoculated into musts, and no clear imposition of one of them was achieved at the end point. Only the commercial QA23 strain dominated at all stages of the fermentation.

### 3.3. Characterization of S. cerevisiae Strains Fermentation Performance

**Figure 5:**
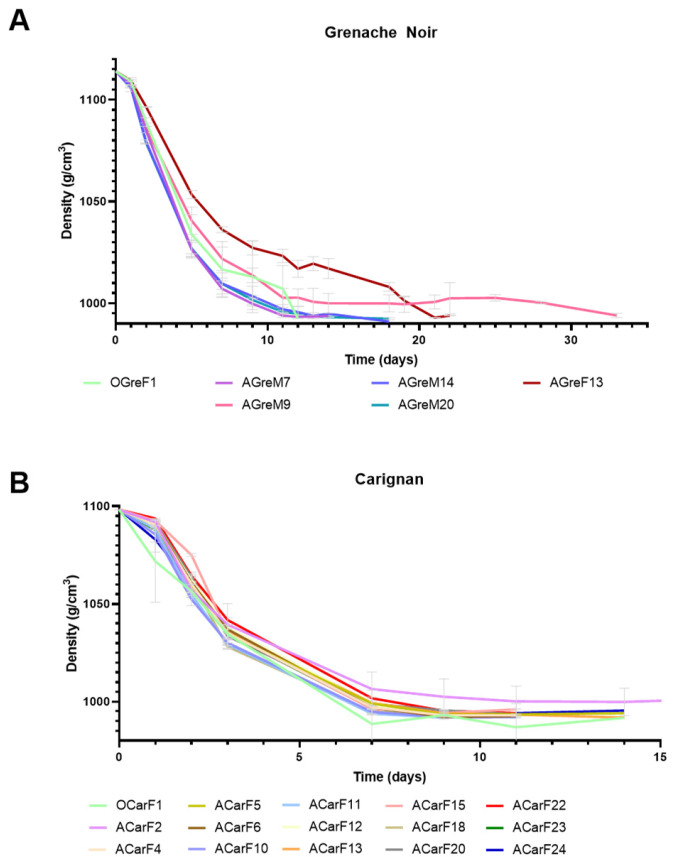
Fermentation performance of strains isolated from grapes at different maturity stages ((**A**) for Grenache Noir and (**B**) for Carignan) and fermentation points, expressed as density. Letters in strain names identify different harvesting states ( O for optimum moment, and A for after the optimum moment), grape varieties (Gre for Grenache Noir and Car for Carignan), and fermentation sampling stages (M for mid-fermentation and F for final fermentation stage). Data are expressed as mean values, and error bars represent standard deviations (*n* = 2).

Single fermentations were performed at a laboratory scale for each of the *S. cerevisiae* strains that were isolated. Different musts with potential alcohol (PA) of 16.0% and 13.7% were used to test the isolated strains in order to simulate the conditions of the musts from each variety (Table 1 and Table A1). Most of the fermentations finished within the first 10–18 days, showing good fermentation performance (Figure 5). In the case of the Grenache must, the only strain isolated from the final fermentation of overripe grapes (AGreF13) had slower fermentation dynamics than those isolated from the grapes at the optimum maturation moment or from the mid-fermentation of the overripe grapes. Furthermore, the AGreM9 strain exhibited an extended phase at the end of the fermentation (Figure 5A). All of the strains isolated from the Carignan grapes presented similar fermentation dynamics, except for ACarF2, which showed slower fermentation (Figure 5B). Other authors have also reported variability in the fermentation dynamics between *S. cerevisiae* strains [56,57].

**Figure 6:**
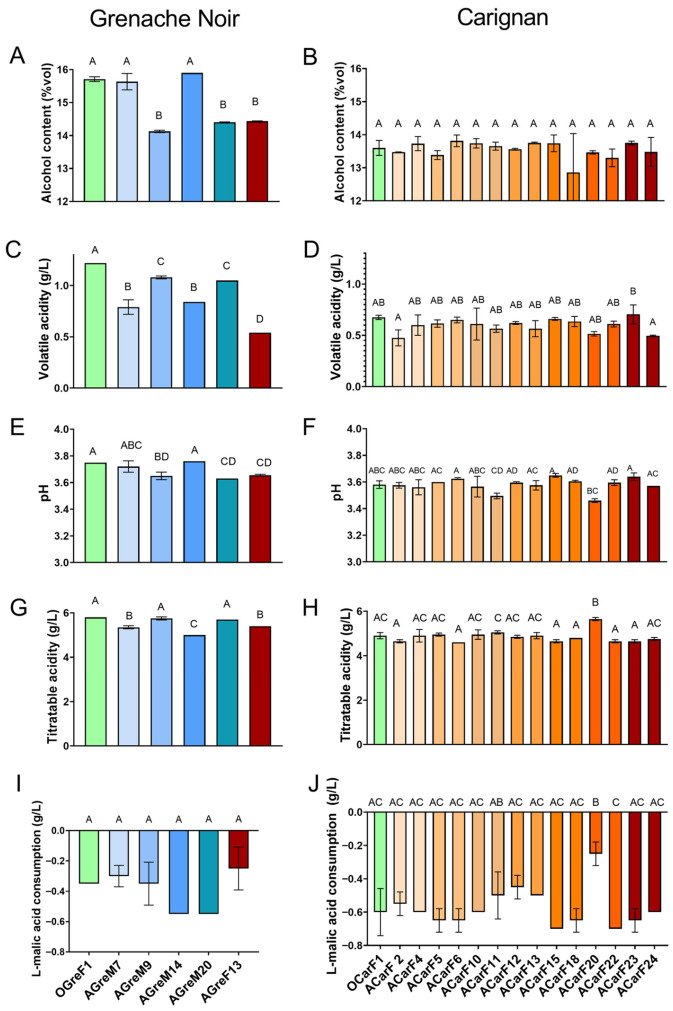
Chemical parameters of wines produced by strains isolated from grapes at different maturity stages for Grenache (**A**,**C**,**E**,**G**,**I**) and Carignan (**B**,**D**,**F**,**H**,**J**) and by fermentation points. (**A**,**B**) alcoholic content, (**C**,**D**) volatile acidity, (**E**,**F**) pH, (**G**,**H**) titratable acidity, and (**I**,**J**) L-malic acid consumption. Data are expressed as mean values (and standard deviation shown as error bars) of *n* = 2 and were analyzed by ANOVA and Tukey HSD post-test (*p* > 0.05). Statistically significant differences are indicated by different letters (A, B, C, D).

The strains isolated from Carignan showed little difference in their ability to convert hexoses to ethanol, with a yield of 0.44–0.47 g/g hexose. The fermented wines obtained ethanol concentrations of 12.86–13.82% *v*/*v* (Figure 6B), which is considered standard for red wines. The strains isolated from Grenache presented lower ethanol yields, ranging between 0.41 and 0.45 g/g hexose and final ethanol concentrations of 13.57–15.90% *v*/*v* (Figure 6A). The strains with slower fermentation dynamics (AGreF13 and AGreM9), were the ones that produced less ethanol, together with AGreM20. Many of the strains in this study have a lower ethanol yield than the mean reported in the literature, which is 0.47 g/g hexose [58,59]. This is particularly relevant, given that the wine industry is currently making significant efforts to find strategies to decrease the final ethanol content of wine. One of these strategies is using strains that produce low ethanol yields [59,60]. It has been described that ethanol yield mainly depends on several fermentation conditions such as temperature or oxygen availability [61,62]. However, it has been reported that the variability among the strains of wild *S. cerevisiae* is low (1.8–3%) [28,58,63]. The strains isolated from Grenache showed greater variability (5.3%) than those from Carignan (1.8%) and than the values previously reported in the literature. Thus, the difference between the Grenache strains was greater than the mean. This reinforces the idea that grapes contain a large heterogeneity of strains and highlights their potential as a good source for finding new yeasts with desirable traits for wine production. In terms of glycerol concentrations, all the strains produced standard yields for wines, and higher than the threshold for sweetness, which is 5.2 g/L of glycerol in white wine [14,64]. Some strains such as OGreF1, OGreF2, and AGreF13, yielded glycerol concentrations ranging 8.6–8.8 g/L, above the average reported for *S. cerevisiae* wine strains (7 g/L) [28,58]. It can be observed that the strains isolated at the final stages of the fermentation, even for the mature or overripe grapes, presented higher glycerol production than those isolated in the middle of the fermentation (8.8 g/L versus 5.45–7.4 g/L). Glycerol is the second most abundant metabolite formed by yeast during the alcoholic fermentation (reviewed in Andorrà et al. [12]). Its production is reported to have a negative correlation with ethanol production (due to redox balancing between the two metabolites) [14,28]. Searching for strains with increased glycerol production could lead to lower ethanol production. This, together with its positive impact on the sweetness of the wine at medium-high concentrations, makes the strains that produce high glycerol of great interest for the oenological industry (reviewed in Goold et al. [14]). In the present study, no correlation has been found between the values of ethanol and glycerol productions, which is supported by the literature; as reported by Monnin et al. [28], the correlation is found mainly in the modified and evolved strains. In terms of the coefficient of variation among strains, in our study, it was 15.8–4.7%, which is lower than that reported by other authors for wine strains (around 10%) [28].

Acetic acid is normally produced by acetic acid bacteria and is measured in terms of volatile acidity. However, it can also be produced by lactic acid bacteria and by yeast during fermentation (reviewed in Bartowsky and Pretorius [65]). Low values of volatile acidity are reported to provide vinegar-like sourness and a nutty and sherry-like aroma. Values exceeding 0.4–0.5 g/L of acetic acid are linked to one of the major off-flavors that come from alcoholic fermentation [66,67]. According to Montsant DO specifications, the permitted concentration of volatile acidity in wines aged less than 1 year is <0.8 g/L for all wines, but it is permitted up to 1.08 g/L for white and rosé and 1.2 g/L for red wines [68]. Most of the wines fermented with strains isolated from Grenache are above those limits, while in the case of Carignan, all are beyond them (Figure 6C,D). Of particular interest are strains ACarF2 and ACarF24, which exhibited the lowest concentrations (above 0.5 g/L), and they would not have aroma considered negative sensorially [12,69]. The strains AGreF13, ACar20, AGreM7, and AGreM9 are also interesting, as they had lower productions than the rest (Figure 6C). For this parameter, the coefficient of variation was 24% for the Grenache strains and 11% for the Carignan ones, following the same trend for ethanol and glycerol, but higher in both cases. These results are supported by the literature, which reports that the diversity in the production of this compound among strains is higher than for ethanol and glycerol production, reporting coefficients of 46% [28,58,61].

In terms of L-malic acid concentration, all the fermented wines presented 1.50–2.05 g/L (Figure 6G,H), which was lower than the concentration of the must (2.25 g/L (Table A1)). During fermentation, the most common pathway of L-malic acid degradation is the malolactic fermentation driven by *Oenococcus oeni*, which produces L-lactic acid. In the present study, the L-lactic acid concentration in the final wines was <0.1 g/L, indicating that malolactic fermentation did not occur or even start during the fermentation process. It has been previously described that some strains of *S. cerevisiae* can both produce or consume this organic acid via the fumarate pathway catalyzed by cytosolic or mitochondrial fumarase, or via the oxaloacetic acid catalyzed by malate dehydrogenase [28,70,71]. Most *S. cerevisiae* wine strains tend to not change or consume low concentrations of this organic acid (<1 g/L or less than 50% of that in the must) [28,69,70,72]. In line with the literature, the present study has shown that the strains of *S. cerevisiae* isolated from Grenache consumed 11–24% of the L-malic acid in the must, while those isolated from Carignan consumed 21–30%. Interestingly, ACarF20 shows 11% consumption, less than the rest of those strains isolated from Carignan.

The strains isolated from the Grenache must OGreF1, AGreM9, and AGreM20 presented high titratable acidity, and this effect was translated to a lower pH value in the latter two strains (Figure 6C,D). In the case of OGreF1, the increase in titratable acidity could be related to the higher volatile acidity (Figure 6C). Moreover, AGreF13 and ACarF11 also presented low pH values. ACarF20 presented the highest titratable acidity among the Carignan strains, resulting in a pH that was 0.19 points lower than the strains with the highest pH (Figure 6E,F). This elevated acidity in ACarF20 could be related to its low consumption of L-malic acid, suggesting a correlation between titratable acidity, pH, and L-malic acid levels [69]. In all cases, the acidity of the final wine increased compared to that of the must.

The main acids in must and wines are tartaric acid, followed by malic, lactic, and succinic acids, which collectively constitute the titratable acidity. This normally remains constant during fermentation [12,69], although it can vary due to the metabolism of microorganisms that can produce or consume some of the acids [12,69]. Due to climate change, wines are generally becoming less acidic as increased sugar concentrations in the must are accompanied by decreased malic acid concentrations [73,74]. The pH affects the microbial stability of wine: the lower the pH (more acidic), the greater the protection against the growth of microorganisms that can produce undesirable compounds. Therefore, there is currently significant research into techniques to obtain wines with lower pH. These techniques include physical technologies such as direct acid addition, ion exchange resins [75], or electromembrane treatments, as well as vineyard management, blending, or the use of microbial and biotechnological resources [70]. L-malic acid in wine is primarily converted by lactic acid bacteria to L-lactic acid, which is a weaker acid that reduces wine acidity. However, this process can also lead to the production of other acids, such as acetic acid, which can impact wine organoleptic properties. Therefore, winemakers monitor this process by maintaining a low pH (pH < 3.30 is recommended to prevent this fermentation [12,69]) and controlling L-malic acid in must and wine to ensure it does not occur inside the bottle [76]. Moreover, the strong acidification of the must, driven by high malic acid concentrations, can have negative consequences on fermentation kinetics [77] and the organoleptic quality of the wine. For this reason, some studies focus on reducing malic acid in wine to shorten malolactic fermentation by limiting the amount of substrate available for degradation [77]. Therefore, the strains identified in the present study that consume L-malic acid are of great interest for controlling wine de-acidification, decreasing acidity in wines with high malic acid concentrations, facilitating malolactic fermentation control, and reducing its duration, as other strains described previously [72].

The screening revealed strains with different production profiles and fermentative dynamics, demonstrating that isolating yeast strains from grapes can help to identify interesting yeasts for the oenological microbiology industry. Therefore, these results are consistent with other studies that report the positive use of autochthonous yeasts for the production of wine. In the case of Grenache, two groups of strains were identified. The first group comprised the AGreF13, AGreM9, and AGreM20 strains, which all exhibited the same pattern: lower ethanol production, lower pH, and higher total acidity. Of particular interest was the AGreF13 strain, which, despite having slow fermentation kinetics, produced less volatile acidity and higher glycerol yields than the rest. The second group included the OGreF1, AGreM7, and AGreM14 strains, which generally exhibited similar behavior: higher alcoholic content, higher pH, and lower ATT, compared to the first group. Moreover, the AGreM7 strain displayed fast fermentation kinetics and lead to a wine with lower volatile acidity and glycerol than the rest, highlighting the potential of this strain for further investigations. For Carignan, the most interesting strain was ACarF20, which decreased pH and volatile acidity, and had the lowest efficiency of L-malic acid consumption. Additionally, strains ACarF24 and ACarF2 produced less volatile acidity; this variability of *S. cerevisiae* strains on volatile acidity production was previously reported in other studies [56]. These results suggest that overripe grapes may harbor strains with greater potential, although further research is needed to draw definitive conclusions.

Interestingly, alcohol, glycerol, and volatile acidity production presented greater variability in the Grenache strains than in the Carignan strains. This could be due to internal heterogeneity, but it may also result from the initial characteristics of the must, as the Grenache must contained a higher sugar concentration. Under these conditions, the strains may face greater ethanol stress, making differences more evident. For this reason, those strains need to be tested in the same must. Thus, a green must was chosen to complement the study, comparing the effects of the strains in different types of musts.

One of the major concerns for the wine industry is the impact of climate change, which, among other effects, increases the sugar content of musts and decreases their acidity, leading to higher ethanol production and lower acidity. Several projects are directed toward identifying the yeast strains that can mitigate these effects in wines [4,10,51,57,73]. For this reason, some strains identified in the present study (AGreF13, AGreM9, ACarF2, and ACarF20) are particularly interesting for the wine industry. Therefore, there is a need to explore the fermentation performance of these strains under conditions that are more similar to those of industrial vinification.

### 3.4. Pilot Scale Fermentations

Pilot scale fermentations were performed using the most interesting *S. cerevisiae* strains isolated from the Grenache and Carignan musts. These were conducted both as single fermentations and as sequential fermentations with the isolated non-*Saccharomyces* strains. Additionally, a control fermentation using the PDM strain was performed.

For *Saccharomyces cerevisiae* strains, a set of promising yeasts, all from overripe grapes, were chosen based on the characterization at laboratory scale: AGreF13 for its low ethanol production and high acidity, ACarF20 for its low capacity to consume malic acid and hence higher acidity, ACarF2 for its low production of volatile acidity, and AGreM7 for both its low production of volatile acidity and its fast fermentation performance. In the oenological research, there is a growing interest in identifying non-*Saccharomyces* strains with interesting properties. Therefore, in this study, we investigated all the non-*Saccharomyces* species found in different musts: *T. delbrueckii* (OGreM1), *H. opuntiae* (ACarM21), *H. uvarum* (BCarM1), and *S. bacillaris* (ACarI7). Previous studies report strains of all those species that have the potential to decrease the ethanol concentrations or improve the aromatic complexity of wines in different ways [48,60,78,79].

Each non-*Saccharomyces* strain was combined with one specific strain of *S. cerevisiae*. Accordingly, the pairs were chosen to potentiate the characteristics of the *S. cerevisiae* strains, as follows: *T. delbrueckii* is one of the most extensively studied non-*Saccharomyces* yeasts, mainly for its low acetic acid and ethanol production compared to *S. cerevisiae*, and it also increases freshness and floral aroma of the wine. Moreover, some strains have been described as high L-malic acid consumers, thereby influencing pH and acidity [48]. This species was combined with ACar20, which showed the highest increase in total acidity and low malic acid consumption in the present article. In the case of *S. bacillaris*, this species is described to increase glycerol production, thereby decreasing the ethanol concentration. There has been substantial research exploring strains with ethanol-reducing potential [60,80], and it was combined with AGre13 to reinforce its potential to decrease ethanol content reported in the laboratory scale fermentations of the present study. For both *Hanseniaspora* spp., this genus is described to enhance aromatic compounds of the wine but has been reported to typically increase volatile acidity [81]. However, this effect is strain-dependent [82], with some strains producing the same or less volatile acidity than some *S. cerevisiae* strains [83,84,85]. For this reason, in the present study, these species were combined with strains ACar2 and AGre7, which both belonged to the group of *S. cerevisiae* strains that produced less volatile acidity.

**Figure 7:**
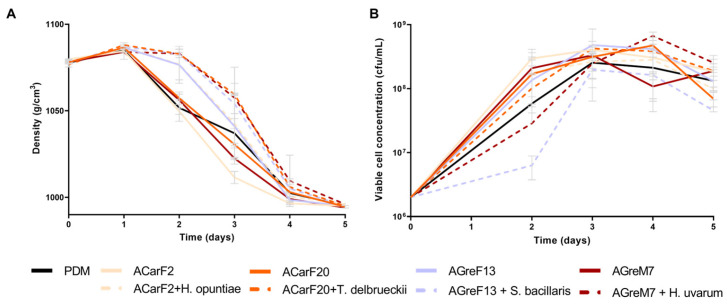
Fermentation performance at pilot scale of wines fermented with different strains. Single fermentations with the *S. cerevisiae* strains, ACarF20, ACarF2, AGreM7, and AGreF13, and mixed fermentations with these *S. cerevisiae* strains sequentially inoculated with strains of the following non-*Saccharomyces* species: *T. delbrueckii*, *H. opuntiae*, *H. uvarum*, and *S. bacillaris*. (**A**) Density. (**B**) Viable cell concentration. Data are expressed as mean values (with standard deviation shown as error bars) of *n* = 2.

The fermentation kinetics of all strains were comparable in duration to the commercial strain PDM and exhibited a higher fermentation rate than those performed at laboratory scale (Figure 5 and Figure 7). In the case of single fermentations, AGreM7 had the highest rate, and GreF13 had the slowest rate (Figure 7A). Furthermore, as described elsewhere [10], the sequential fermentations had a longer lag phase: during the first 48 h, where only the non-*Saccharomyces* strains were present, there was a minimal decrease in density. Once the *S. cerevisiae* strain was inoculated, the fermentation proceeded normally, following the same pattern as the single fermentation and finishing at the same time. Surprisingly, the single fermentation of strain AGreF13 had the same kinetics as the fastest of the mixed fermentations, namely, that of *H. opuntiae* with ACarF2. In terms of population dynamics, all the strains behaved in the same way, increasing the population to values around 10^8^ cfu/mL. However, *S. bacillaris* and *H. uvarum* had slower growth, reaching 10^7^ cfu/mL after two days of fermentation (Figure 7B). After *S. cerevisiae* was inoculated, all the fermentations had populations around 10^8^ cfu/mL. These results correlated with the density measurement, since these were the fermentations with the longest lag phases.

**Figure 8:**
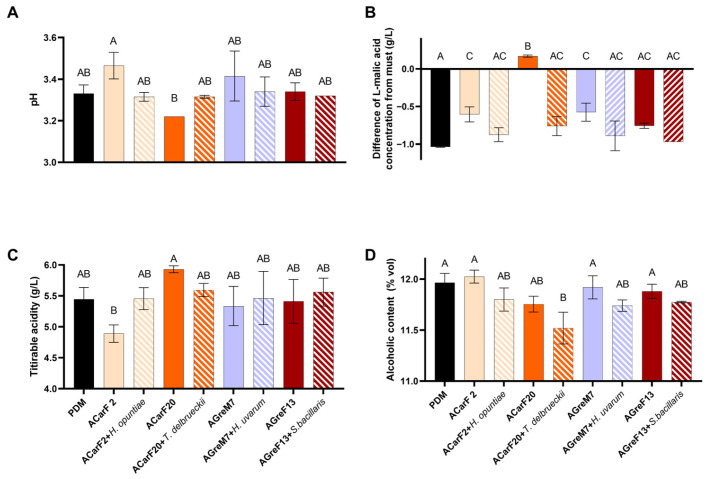
Chemical composition of wines produced by strains isolated from different maturity stages of grapes and by fermentation points. Single fermentations with the *S. cerevisiae* strains ACarF20, ACarF2, AGreM7, and AGreF13 and mixed fermentations with these *S. cerevisiae* strains sequentially inoculated with strains of the following non-*Saccharomyces* species: *T. delbrueckii*, *H. opuntiae*, *H. uvarum*, and *S. bacillaris*. (**A**) pH; (**B**) L-malic acid reduction from must to wine; (**C**) titratable acidity; and (**D**) alcoholic content. Data are expressed as mean values (with standard deviation shown as error bars) of *n* = 2 and were analyzed by ANOVA and Tukey HSD post-test (*p* > 0.05). Statistically significant differences are indicated by different letters (A, B, C).

In all cases, ethanol production was higher than expected based on the potential alcohol of the must (Figure 8D; Table A1). The results reported a yield of 0.53 g/g hexose for the commercial strain and between 0.50 and 0.52 g/g hexose for the isolated strains, which was higher than that of the lab scale. This higher yield could be attributed to differences in must sugar concentration, as the must used for the pilot-scale fermentation had a lower sugar concentration (Table A1). Furthermore, the scale change might have affected these outcomes. Compared to their respective musts, all *S. cerevisiae* strains produced tendentially less ethanol than commercial control. This effect was more pronounced in strains ACarF20 and AGreF13 (Figure 8D), with the latter showing similar behavior at laboratory scale. In all mixed fermentations, the ethanol content was slightly lower, which is in line with the literature reporting that some strains of these species can produce decreases in ethanol content [60,70,86]. The *T. delbrueckii* case is particularly noteworthy, as the mixed fermentation with *S. cerevisiae* showed a decrease of 0.25% *v*/*v* compared to the single fermentation of *S. cerevisiae*. This decrease was significant (0.45% *v*/*v*) compared to the commercial strain (Figure 8D). The previous literature highlights that some strains of *T. delbrueckii* in sequential fermentations with *S. cerevisiae* produce a decrease in ethanol content compared to single fermentations with *S. cerevisiae* (from 0.15 to 0.5 *v*/*v*) [75,87,88,89,90,91]. Therefore, in the present study, the synergistic effect of the two indigenous strains resulted in a significant decrease in ethanol content.

No differences in volatile acidity were found between the different strains, neither for *S. cerevisiae* nor for the non-*Saccharomyces* yeasts. The values were between 0.25 and 0.34 g/L, which were lower than the legal limits and industry preferences and lower than the results at laboratory scale. The *Hanseniaspora* spp. strains are particularly noteworthy. While this genus was previously described to produce high amounts of acetic acid, it has been recently shown that this production is strain-dependent, with some strains increasing, decreasing, or having no effect on acetic acid levels (reviewed in Tufariello et al., 2021) [78]. Notably, *Hanseniaspora* spp. strains isolated in the present study did not alter volatile acidity.

L-malic and L-lactic acid concentrations were measured at the end of fermentation. In all cases—except for ACarF20—the L-malic acid concentration decreased by 0.17–1.04 g/L, resulting in final wine concentrations of 0.22–1.36 g/L (Figure 8B). As observed in laboratory-scale fermentations, L-lactic acid remained <0.1 g/L, indicating that yeasts consumed malic acid without malolactic fermentation occurring. The *S. cerevisiae* strains ACarF2, AGreM7, and AGreF13 consumed L-malic acid to a lesser extent than the commercial control PDM (Figure 8B). In general, the consumption was higher than at the laboratory scale (at pilot scale, percentages were around 40–80%; at laboratory scale, they were 11–28%). This can be related to the lower must concentration of L-malic acid (1.22 g/L compared to 2.25 g/L in laboratory-scale must, Table A1), the scale effect, or the presence of other microorganisms in the must. Moreover, strain ACarF20 showed no L-malic consumption, following the same trend as observed at the laboratory scale, where it consumed less than the rest. The mixed fermentations consumed slightly more of this acid than the single fermentations, with this difference being significant in the case of *T. delbrueckii* (Figure 8B). The previous literature shows that this species can either produce or consume L-malic acid in a strain-dependent manner. In sequential fermentations with *S. cerevisiae*, moderate malic acid consumption has been observed in the previous literature (20–25% of this acid) [48,69,92]. The trend in L-malic acid consumption observed for *S. bacillaris* and *Hanseniaspora* spp. is supported by the literature [69,70,86,93]. Moreover, fermentation with *T. delbrueckii* is reported to have a positive influence on malolactic fermentation [94,95], while strains of *S. bacillaris*, *H. uvarum*, and *H. vineae* could negatively affect malolactic fermentation performance [53,94].

In terms of pH and titratable acidity, no significant differences were observed when compared to wines fermented with the control strain (Figure 8A,C). ACarF20 exhibited one of the lowest pH and highest acidity values, while ACarF2 followed the opposite pattern: higher pH and thus lower acidity. Accordingly, ACarF20 had a pH 0.25 units lower than ACarF2 and 1.04 g/L higher titratable acidity, similar to the effect observed at laboratory scale, where the differences were 0.12 pH units and 1.00 g/L of titratable acidity (Figure 8A,C). This could be correlated with the fact that L-malic acid was not consumed by ACarF20 and remained available for conversion to L-lactic acid. This could result in higher total acid concentration in the final wine compared to the other conditions, thus leading to higher acidity. Mixed fermentations exhibited slightly lower pH than single fermentations, with the exception of ACarF20—*T. delbrueckii* pairing, where pH increased and acidity decreased likely due to *T. delbrueckii* consumption of L-malic. A review by Benito et al. [48] reports that some strains of *T. delbrueckii* can decrease acidity. The mixed fermentations in the present article had values more similar to those of the control, suggesting that the presence of non-*Saccharomyces* strains mitigates the effect of the *S. cerevisiae*. In the case of *H. uvarum*, it produces a trend toward pH reduction and increased acidity. This is supported by Thivijan et al. [93], who showed that some strains of this species can consume malic acid, thereby decreasing wine pH.

The results of the pilot-scale fermentations reinforce those obtained at laboratory scale. The indigenous strains isolated in this article have shown good fermentation capacity compared to the commercial strain PDM, or even better performance in the case of ACarF2. It is important to highlight that these strains produce less ethanol than the control strain. When combined with non-*Saccharomyces* strains (mainly *H. uvarum* and *T. delbrueckii*), ethanol reductions exceeding 0.4% were achieved, addressing a critical challenge in the modern oenology industry. Moreover, these strains do not result in a higher pH than the control, which could otherwise compromise the stability of the wine, and some strains (ACarF2) even decrease pH until 3.22 while simultaneously increasing total acidity.

### 3.5. Fermentative Aromas

**Figure 9:**
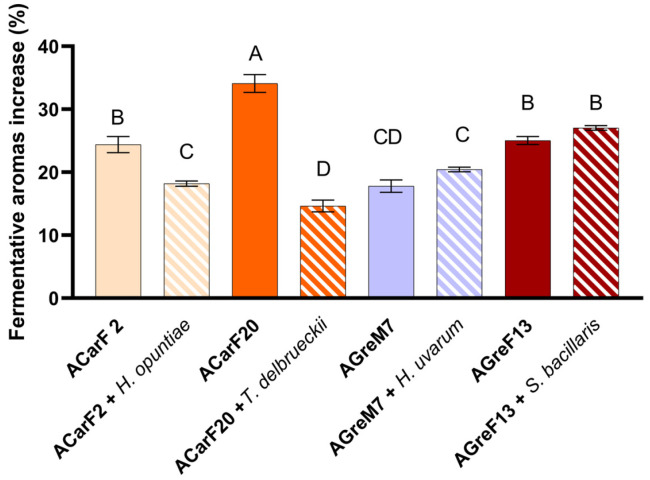
Increase in fermentative aromas of wines compared to the commercial control (PDM). Single fermentations with the *S. cerevisiae* strains ACarF20, ACarF2, AGreM7, and AGreF13 and mixed fermentations with these *S. cerevisiae* strains sequentially inoculated with strains of the following non-*Saccharomyces* species: *T. delbrueckii*, *H. opuntiae*, *H. uvarum*, and *S. bacillaris*. Data are expressed as mean value (and standard deviation in the error bar) of *n* = 2 by ANOVA and Tukey HSD post-test (*p* > 0.05), and statistically significative differences are indicated by different letters (A, B, C, D).

**Figure 10:**
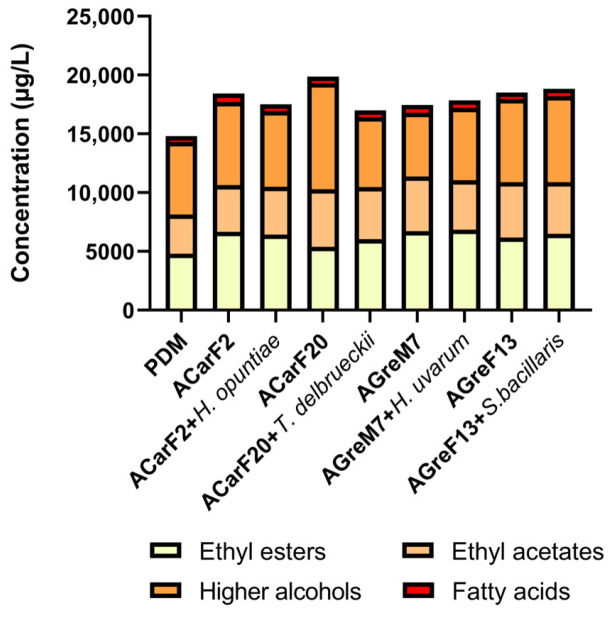
Total fermentative aroma concentrations for each family after alcoholic fermentation for all the studied conditions. Single fermentations with the *S. cerevisiae* strains ACarF20, ACarF2, AGreM7, and AGreF13 and mixed fermentations with these *S. cerevisiae* strains sequentially inoculated with strains of the following non-*Saccharomyces* species: *T. delbrueckii*, *H. opuntiae*, *H. uvarum*, and *S. bacillaris*. Data are expressed as mean values; the statistics are reflected in Table 2.

Analysis of the fermentative compounds revealed that the indigenous strains induced a general increase. Among the *Saccharomyces* strains, ACarF20 showed the highest increase in fermentative aroma compounds compared to PDM (+34.1%, Figure 9), being significantly higher than all the other strains. This effect was mainly due to enhanced synthesis of alcohols and acetates, with ethyl acetate and ethyl isovalerate being particularly relevant for their contribution to fruity notes [75] (Figure 10; Table 2). Strains AGreF13 and ACarF2 showed intermediate increases in similar magnitude, ranging between 24.4 and 27.0%, which were maintained in the sequential fermentation with *S. bacillaris*. A third group, comprising ACarF2 + *H. opuntiae* and AGreM7 (both single and sequentially inoculated with *H. uvarum*), displayed more moderate increases, ranging from 17.8 to 20.5%. Finally, ACarF20 + *T. delbrueckii* exhibited the lowest increase of +14.7%, associated with limited production of higher alcohols, especially isobutanol and isoamyl alcohol (Table 2). Overall, these results demonstrate that ACarF20 is the most effective strain for promoting fermentative aroma production compared to the control treatment, although this effect is notably diminished when sequentially inoculated sequentially with *T. delbrueckii*. Fernandes et al., 2021 reviewed that the production of fermentative aroma by *T. delbrueckii* is strain-dependent [92]; the results presented in the present article align with those studies.

When comparing pure *Saccharomyces* fermentations with sequential inoculation trials involving non-*Saccharomyces* yeasts, different behaviors were observed (Figure 9). For ACarF20, sequential inoculation with *T. delbrueckii* resulted in the lowest increase in fermentative compounds (14.7%), which was 19.4% less than the pure strain alone. A similar trend was observed for ACarF2, where sequential inoculation with *H. opuntiae* led to a 6.3% decrease in fermentative aromas relative to the pure culture. However, this mixed fermentation still showed an 18.2% increase compared to the control condition using the PDM yeast. In both cases, the reductions in fermentative aroma concentrations were statistically significant. Conversely, AGreF13 and AGreM7 showed comparable concentrations under both conditions, with no significant differences between pure and mixed fermentations. Despite the lack of statistical significance, sequential inoculation resulted in a slight increase, ranging from 2.0 to 2.7%.

Figure 10 shows the total concentration of major volatile compound families (total ethyl esters, total ethyl acetates, total higher alcohols, and total fatty acids) obtained from different fermentation treatments. The control condition (PDM) exhibited moderate levels of esters and alcohols, with relatively low concentrations of fatty acids. All inoculated fermentations showed an increase in at least one group of volatile compounds compared to the control treatment, indicating an active contribution of the inoculated strains to aroma compound formation.

Among the pure cultures, ACarF20 displayed the highest production of higher alcohols, significantly exceeding all other treatments, which suggests a strong fermentative activity and active synthesis of alcohols. Notably, this strain is the one that produces the lowest ethanol concentration. The other single fermentations with ACarF2, AGreM7, and AGreF13 also produced high levels of total esters, together with intermediate levels of alcohol and very low levels of fatty acids. Fatty acid concentrations were below the OAV threshold. The presented results represent a balanced pattern typical of *Saccharomyces cerevisiae* reported in the literature [56,96,97,98].

In mixed fermentations involving non-*Saccharomyces* yeasts, each species showed a different pattern. *H. opuntiae* and *H. uvarum* showed a decrease in fatty acids, as reported by Tufariello et al. [78] while also decreasing or maintaining ethyl acetates and ethyl esters levels (Figure 10). Therefore, these *Hanseniaspora* species produced wines with the most balanced and desirable volatile profiles. For *T. delbrueckii*, the strain isolated in the present study increased ethyl esters levels while decreasing higher alcohol concentrations (Figure 10). This behavior was consistent with other studies reviewed by Benito et al., Tufariello et al., and Maicas et al. [48,78,79]. Similarly, the *S. bacillaris* strain increased the levels of higher alcohols, ethyl esters, and fatty acids, consistent with findings reviewed by Tufariello et al. [78]. These results confirm that mixed fermentations combining *Saccharomyces* and non-*Saccharomyces* yeasts isolated in the present study can improve the aromatic balance and sensory equilibrium of wines.

### 3.6. Sensorial Analysis

The sensory evaluation of pilot-scale wines fermented with different *Saccharomyces cerevisiae* strains—either as pure cultures or in mixed fermentations with non-*Saccharomyces* yeasts (*T. delbrueckii*, *H. uvarum*, *H. opuntiae*, and *S. bacillaris*)—revealed significant strain-dependent effects on the aromatic and gustatory properties of the wines. No significant differences (*p* < 0.05) were observed in color intensity among conditions. These findings confirm that yeast metabolism exerts only a limited effect on phenolic extraction and wine chromaticity, as reported in the literature [16].

**Figure 11:**
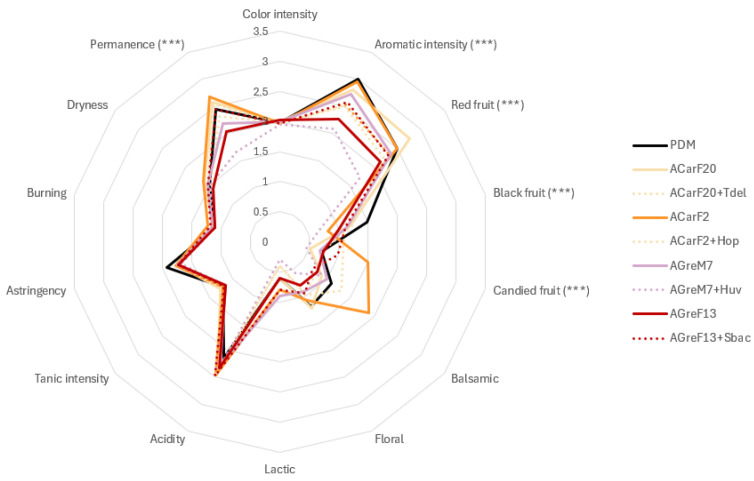
Radar chart of the color, aromatic, and gustatory profiles of the wines obtained in pilot-scale microvinifications. The asterisks symbol indicates statistically significant differences between different wines, analyzed by ANOVA and Tukey HSD post-test (*p* < 0.05).

Aromatic analysis showed clear differentiation among fermentations (Figure 11). The wines produced using strains ACarF20 and ACarF2 displayed the highest aromatic intensity, which was comparable to that of the commercial control. ACarF20 was particularly associated with intense red-fruit notes, whereas ACarF2 enhanced candied-fruit descriptors, contributing to a riper and sweeter aromatic profile. These aromatic differences could be attributed to the high concentration of ethyl esters (Table 2). In ACarF20, the fruity profile was particularly correlated with increased ethyl isovalerate levels. These results indicate that strain-specific modulation of secondary metabolism, particularly within esterification and higher-alcohol pathways, governs the sensory differentiation, which was observed in the literature [99,100]. The enhanced fruity character of ACarF20 may reflect higher β-glucosidase activity or differential release of bound primary aroma precursors, mechanisms previously reported in fermentations involving non-*Saccharomyces* species [81]. Interestingly, the *H. uvarum* strain isolated in the present study produced wines with lower aromatic intensity and reduced red- and black-fruit aromas compared to the single fermentations with *S. cerevisiae*.

Mixed fermentations showed favorable synergistic effects. Sequential inoculation with *T. delbrueckii* or *S. bacillaris* produced wines with aromatic and structural profiles similar to or exceeding those of pure *S. cerevisiae* fermentations (Figure 11, Table 2). The coexistence of *S. cerevisiae* with non-*Saccharomyces* yeasts has been reported to enhance volatile diversity and aromatic complexity through complementary metabolic interactions, such as increased synthesis of esters [74,101]. In the present study, the maintenance of aromatic intensity and balance indicates that population dynamics between species were compatible and did not result in metabolic suppression.

Differences in gustatory parameters corroborated the sensory data. Wines fermented with ACarF2 exhibited a significantly higher attack volume (*p* < 0.05) (Figure 11). Fermentations with *T. delbrueckii*, *H. opuntiae*, and *S. bacillaris* also showed elevated scores, suggesting their ability to enhance mouthfeel and structural balance. Unctuosity followed a similar pattern, with ACarF2 achieving the highest value, while ACarF20 and the mixed fermentation ACarF2 + *H. opuntiae* remained statistically comparable to the control. These improvements are likely linked to increased glycerol formation and mannoprotein release, both of which are previously reported to contribute to viscosity and roundness [16,56]. Figure 11 indicates that mouth permanence varied slightly among treatments, but several fermentations, including ACarF2 (both pure and sequential inoculated with *H. opuntiae*) ACarF20, and AGreF13 + *S. bacillaris*, achieved scores equivalent to the control.

The ability of ACarF20 to reduce ethanol levels and increase acidity without compromising sensory acceptance represents a biotechnological approach to addressing challenges imposed by climate-driven grape over-ripeness. Under modified compositional conditions, the maintenance of the organoleptic harmony highlights the potential of wild strains to stabilize acidity and freshness. Those are traits increasingly desired in warm-climate viticulture [56,101]. These findings align with previous reports identifying selected *S. cerevisiae* strains as effective tools for natural alcohol reduction while maintaining sensory balance [100,101].

## 4. Conclusions

The present study highlights the crucial role of grape harvest timing in shaping the microbial populations present on the berries and, consequently, influencing their fermentative capacity and metabolite production. The observed variations across the ripening stages suggest that certain harvest moments may favor a more balanced microbial ecology and improved fermentation performance. These findings reinforce the importance of considering microbiological criteria, alongside traditional measures of technological and phenolic maturity, when determining the optimum harvest date. This optimum ripeness stage is essential, especially in the current context where many wineries rely on spontaneous fermentation for wine production to better express their terroir. Integrating these parameters could lead to wines with greater consistency, complexity, and typicity under varying viticultural conditions.

As the grapes ripen, we have observed that *Saccharomyces* species become more prevalent on the skins, at the expense of molds and non-*Saccharomyces* species. Therefore, ensuring the presence of *Saccharomyces* yeasts provides greater assurance of successful spontaneous fermentation initiation and reduces the risk of premature fermentation arrest. The species isolated in the present study exhibit several characteristics that are beneficial for overcoming the challenges faced by the wine industry, such as low sugar-to-ethanol yield, high L-malic acid consumption and acidity, and increased production of volatile compounds. These characteristics were obtained without affecting the sensorial properties when compared to a commercial strain.

The overall sensory evaluation favored wines fermented with the ACarF20 and ACarF2 strains, as well as those fermented with the *T. delbrueckii*, *H. opuntiae*, and *S. bacillaris* strains. All of these strains maintained the desirable characteristics of the *S. cerevisiae* strain in the mixed fermentation. These outcomes confirm that indigenous *S. cerevisiae* strains, either on their own or alongside non-*Saccharomyces* yeasts, can produce wines with an equal or superior sensory balance, while also influencing key compositional parameters. Their application reinforces the concept of “microbial terroir,” highlighting the oenological and ecological value of regional yeast biodiversity. The utilization of such biodiversity contributes to sustainable winemaking practices by reducing reliance on commercial starter cultures and promoting wines with distinctive regional identity. These results provide a first insight on the evolution of grape microbiome in the Carignan and Grenache Noir varieties of Montsant DO. They lay the groundwork for further research in this topic in order to extend the study in several directions: across the entire production area, in other varieties, and under different climatic conditions.

This study unravels two important implications. First, it provides guidance on isolation strategies for different yeast types. If *Saccharomyces* species are desired, more overripe grapes should be harvested. Conversely, if non-*Saccharomyces* yeasts are of interest, less ripe grapes are preferable. Second, the study demonstrated that the yeast performing fermentation can be decoupled from the grape maturation stage. Specifically, microorganisms can be isolated from one maturation point and inoculated in a must from different ripeness. This allows the potential use of a yeast strain independently of the microbiota of the grape. This approach would be particularly useful for different applications, such as the harvest of underripe grapes to obtain wines with lower ethanol content.

## Figures and Tables

**Table 2:** Concentration of fermentation markers by families for all studied wines (µg/L). Data are expressed as mean value (standard deviation) of *n* = 2 analyzed by ANOVA and Tukey HSD post-test (*p* < 0.05), and statistically significant differences between rows are indicated by different letters (a, b, c, d, e, f, g, h, i).

	PDM	ACarF20	ACarF20 + *T. delbrueckii*	ACarF2	ACarF2 + *H. opuntiae*	AGreM7	AGreM7 + *H. uvarum*	AGreF13	AGreF13 + *S. bacillaris*
Ethyl butyrate	15 ± 0.01 e	154 ± 1.66 a	25 ± 0.21 c	20 ± 0.21 d	25 ± 0.09 c	26 ± 0.21 c	22 ± 0.06 d	26 ± 0.14 c	29 ± 0.09 b
Ethyl isovalerate	3 ± 0.00 b	132 ± 1.42 a	3 ± 0.03 b	3 ± 0.04 b	4 ± 0.01 b	4 ± 0.03 b	4 ± 0.01 b	3 ± 0.02 b	3 ± 0.01 b
Ethyl hexanoate	1168 ± 1.1 f	1190 ± 12.8 f	1422 ± 11.60 de	1523 ± 16.04 bc	1413 ± 5.40 e	1519 ± 12.69 c	1583 ± 4.67 a	1454 ± 7.78 d	1561 ± 4.60 ab
Ethyl octanoate	2435 ± 2.3 f	2668 ± 28.7 e	3201 ± 26.11 d	3555 ± 37.43 a	3434 ± 13.08 b	3577 ± 29.87 a	3637 ± 10.72 a	3320 ± 17.7 c	3429 ± 10.20 b
Ethyl decanoate	390 ± 0.37 h	623 ± 6.7 g	722 ± 5.89 f	859 ± 9.05 c	886 ± 3.38 b	801 ± 6.69 d	945 ± 2.79 a	763 ± 4.08 e	777 ± 2.29 e
Ethyl dodecaonate	13 ± 0.01 i	15 ± 0.16 h	46 ± 0.38 d	51 ± 0.54 c	62 ± 0.24 b	39 ± 0.33 e	64 ± 0.19 a	18 ± 0.1 g	30 ± 0.09 f
Diethyl succinate	782 ± 0.75 a	607 ± 6.53 de	608 ± 4.96 de	651 ± 6.85 c	620 ± 2.37 d	748 ± 6.25 b	599 ± 1.77 e	603 ± 3.23 e	640 ± 1.89 c
Total esthers	4805 ± 4.60 g	5389 ± 57.98 f	6027 ± 49.17 e	6663 ± 70.16 bc	6434 ± 24.57 d	6714 ± 56.07 ab	6854 ± 20.21 a	6187 ± 33.11 e	6498 ± 19.16 cd
Ethyl acetate	2968 ± 2.84 e	4101 ± 44.12 a	3885 ± 31.69 b	3494 ± 36.8 d	3459 ± 13.21 d	4125 ± 34.45 a	3657 ± 10.78 c	4027 ± 21.6 a	3696 ± 10.9 c
Isobutyl acetate	19 ± 0.02 i	80 ± 0.86 a	37 ± 0.3 c	28 ± 0.3 f	30 ± 0.12 e	22 ± 0.18 h	26 ± 0.08 g	40 ± 0.21 b	33 ± 0.1 d
Isoamyl acetate	344 ± 0.33 h	703 ± 7.57 a	518 ± 4.23 e	448 ± 4.71 g	550 ± 2.1 d	492 ± 4.11 f	510 ± 1.5 e	618 ± 3.31 c	649 ± 1.91 b
2-phenylehtyl acetate	7 ± 0.01 g	7 ± 0.08 f	7 ± 0.06 g	9 ± 0.09 b	8 ± 0.03 d	8 ± 0.06 e	6 ± 0.02 h	9 ± 0.05 c	9 ± 0.03 a
Total ethyl acetates	3338 ± 3.20 f	4891 ± 52.63 a	4447 ± 36.28 c	3979 ± 41.90 e	4048 ± 15.46 e	4647 ± 38.81 b	4199 ± 12.38 d	4693 ± 25.12 b	4388 ± 12.94 c
Isobutanol	622 ± 0.6 f	1746 ± 18.79 a	809 ± 6.6 c	765 ± 8.05 d	688 ± 2.63 e	511 ± 4.27 h	560 ± 1.65 g	839 ± 4.49 b	775 ± 2.29 d
Isoamyl alcohol	5406 ± 5.18 e	7147 ± 76.9 a	5018 ± 40.93 f	6093 ± 64.16 c	5657 ± 21.61 d	4773 ± 39.87 g	5501 ± 16.22 de	6117 ± 32.7 c	6426 ± 18.95 b
Benzyl alcohol	43 ± 0.04 b	39 ± 0.42 c	26 ± 0.21 g	53 ± 0.56 a	26 ± 0.1 g	52 ± 0.44 a	30 ± 0.09e	33 ± 0.17 d	28 ± 0.08 f
2-phenylethyl alcohol	95 ± 0.09 b	94 ± 1.01 b	71 ± 0.58 d	136 ± 1.43 a	70 ± 0.27 d	78 ± 0.65 c	55 ± 0.16 e	71 ± 0.38 d	77 ± 0.23 c
Alcohols	6166 ± 5.91 e	9026 ± 97.12 a	5923 ± 48.32 f	7046 ± 74.19 c	6442 ± 24.60 d	5414 ± 45.22 g	6147 ± 18.12 e	7060 ± 37.79 c	7307 ± 21.54 b
Hexanoic acid	386 ± 0.37 d	390 ± 4.19 d	467 ± 3.81 c	500 ± 5.27 b	469 ± 1.79 c	503 ± 4.2 b	531 ± 1.57 a	476 ± 2.55 c	521 ± 1.54 a
Octanoic acid	100 ± 0.1 de	134 ± 1.44 c	100 ± 0.82 d	205 ± 2.16 a	88 ± 0.34 f	150 ± 1.25 b	97 ± 0.28 de	86 ± 0.46 f	96 ± 0.28 e
Decanoic acid	17 ± 0.02 g	27 ± 0.3 b	19 ± 0.15 ef	35 ± 0.37 a	21 ± 0.08 d	24 ± 0.2 c	19 ± 0.06 e	18 ± 0.1 f	5 ± 0.02 h
Fatty acids	503 ± 0.48 g	552 ± 5.93 f	586 ± 4.78 e	740 ± 7.79 a	578 ± 2.21e	677 ± 5.66 b	647 ± 1.91 c	580 ± 3.11 e	622 ± 1.83 d

## Data Availability

The original contributions presented in this study are included in the article. Further inquiries can be directed to the corresponding author.

## Appendix A

**Table A1:** Chemical composition of musts used for fermentations of laboratory and pilot scale. PA stands for potential alcohol, ATT for total titratable acidity, PAN for Primary Amino Nitrogen, and YAN for yeast assimilable nitrogen.

Must	° Brix	PA (% *v*/*v*)	Density (g/cm^3^)	ATT (g/L)	pH	PAN	NH_4_	NFA	L-Malic Acid (g/L)
Laboratory scale, Grenache Noir	23.9	14.06	1098	3.3	3.69	127	88.5	196	2.3
Laboratory scale, Carignan	27	15.98	1114	3.3	3.69	127	88.5	196	2.25
Pilot scale	19	10.73	1.078	6.0	3.357	73.4	42.6	107	1.2245
